# Associations Between Parental Physical Activity and Preschool Children’s Physical Activity and Social Behavior: A Cross-Sectional Study

**DOI:** 10.3390/children13060763

**Published:** 2026-05-30

**Authors:** Despoina Ourda, Maria Karatzioti, Marianthi Koutsokosta, Athanasios Gregoriadis, Vassilis Barkoukis

**Affiliations:** 1 Department of Physical Education and Sport Science, Aristotle University of Thessaloniki; 54124 Thessaloniki, Greece; despoino@phed.auth.gr (D.O.); mar_rg23@hotmail.com (M.K.); mkoutsoko@phed.auth.gr (M.K.); 2 Department of Early Childhood Education, Aristotle University of Thessaloniki, 54124 Thessaloniki, Greece; asis@nured.auth.gr; 3 Department of Life and health Sciences, Frederick University, 1036 Nicosia, Cyprus

**Keywords:** kindergartner, parental influence, parental modeling, antisocial behavior, prosocial behavior

## Abstract

**Highlights:**

**What are the main findings?**

- Parental physical activity is significantly associated with multiple levels of children’s physical activity.
- Parental physical activity was not significantly associated with any of the children’s psychosocial outcomes.

**What are the implications of the main findings?**

- Interventions should prioritize structured and unstructured family-based weekend activities.
- Intervention programs should emphasize parental modeling, shared activity time, and parental physical activity habits as key mechanisms for increasing children’s physical activity.

**Abstract:**

Background/Objectives: This study examined the association between parental physical activity and preschool children’s physical activity and social behavior. Methods: Participants were 151 preschool children (70 girls, 81 boys; Mage = 52.51 months, SD = 3.38) attending public and private kindergartens in Thessaloniki (Greece) and Nicosia (Cyprus). Children’s psychosocial development was assessed by kindergarten teachers using the Strengths and Difficulties Questionnaire, while parents reported their own and their children’s physical activity through the Preschool-age Children’s Physical Activity Questionnaire (home version). Descriptive statistics and Pearson correlations were computed, and the main hypotheses were tested using multiple linear regression analyses. Results: Results indicated a consistent positive association between parental physical activity and children’s physical activity across intensity levels. Parental physical activity frequency and duration during both weekdays and weekends was significantly associated with children’s low-, moderate-, and vigorous-intensity physical activity, while parental beliefs about physical activity were negatively associated with children’s sedentary behavior. In contrast, parental physical activity showed no significant association with all indicators of social behavior at school, including emotional symptoms, conduct problems, hyperactivity/inattention, and prosocial behavior. Conclusions: Overall, the findings support the role of parental physical activity as an important correlate of preschool children’s physical activity behavior, while its direct association with broader psychosocial development appears small. These results highlight the importance of parental role modeling and attitudes toward physical activity, particularly in shaping children’s movement behaviors.

## 1. Introduction

Research evidence has extensively acknowledged the association between consistent engagement in physical activity (PA) and benefits for an individual’s psychosocial development and mental health [1,2]. Despite the abundance of empirical evidence linking physical activity to positive outcomes, a significant proportion of the population fails to comply with the established national guidelines. Yao and Rhodes [3] have noted that a significant number of children are not engaging in sufficient levels of physical activity to reap the benefits associated with regular participation in physical activity. This is also true for younger children [4,5]. During the preschool years, psychosocial development plays a crucial role in shaping the future growth trajectory of children [6]. According to Tzavara et al. [7], the physical activity levels of children may be significantly influenced by the quality of their relationships with their parents and the environment in which they live. Nevertheless, the effect of parental physical activity on preschool children’s social behavior and physical activity participation has not been adequately investigated in the literature.

According to Cosso et al. [8], parents are significant agents capable of exerting a profound impact on the psychosocial development of their children, whereas Harwood et al. [9] asserted their impact on the engagement of their children in physical activities. Parental influence on preschool children’s psychosocial development and physical activity participation has been a subject of research interest. The characteristics of family members, as explored by Ryan [10], also impact children’s academic performance and conduct. For instance, Sigmundova et al. [11] reported that father–son pairs were more active than mother–son pairs. Also, children with active mothers were more likely to meet physical activity recommendations. Moreover, parents’ physical activity had an indirect influence on children’s physical activity through parental support, specifically by facilitating transportation to activity opportunities [12]. In addition, parental beliefs and expectations regarding their children’s success influence socialization practices, including engagement in physical activity [13]. The importance of family and kindergarten connections in facilitating observational learning and behavior replication has also been highlighted by O’Connor et al. [14] and Minh et al. [15].

Empirical studies suggest that engagement in sports and physical activity contributes to children’s well-being and the development of healthy habits [6,16]. Moreover, research has examined the influence of parental physical activity on the social interactions of children, revealing associations between the recreational activities of parents and the peer relationships of their kids. According to Pyper et al. [17], the provision of parental support, encouragement, and facilitation of outdoor activities involving peers and family members are additional factors that contribute to the development of healthy peer relationships. Parental behavior can influence their offspring, increasing the likelihood of emulation [18]. Weekday leisure activities exhibited a more favorable impact due to a greater range of available options [15]. Overall, the family context, along with various contextual factors such as neighborhood and school environments, influences a child’s holistic development [15].

The influence of parents on their children’s physical activity levels has been observed across various indicators. Existing evidence suggests that the family environment plays a crucial role in children’s physical activity [15]. Research by Timperio et al. [19] highlights the impact of the family environment on children’s physical activity. Oliver et al. [20] and Fuemmeler et al. [21] found associations between parental and children’s physical activity levels. In particular, Fuemmeler et al. [21] found a positive correlation between parental sedentary behavior on weekends and sedentary behavior in their children. Schoeppe et al. [18] demonstrated that children with active parents were more likely to engage in physical activity.

Existing literature shows a positive correlation between parental physical activity levels and the physical activity participation of preschool-aged children [22]. Parents can effectively promote their children’s physical activity by engaging in physical activity themselves, participating in active play, and visiting venues that offer physical activity opportunities [22,23]. Furthermore, parental support behaviors have been found to significantly affect children’s physical activity levels. Supportive parenting behaviors, such as facilitating transportation to physical activity locations, promoting family meals without television, fostering outdoor activity with friends and family, and engaging in physical activity with children, positively impact children’s opportunities for physical activity [17]. Spending more time outdoors and having access to diverse play areas also contribute to higher levels of physical activity in children [17]. The broader context of the family and community environment, including factors like equipment availability, financial resources, logistical assistance, emotional support, and parental modeling, positively influences children’s physical activity levels [19].

### The Present Study

Literature consistently demonstrates an association between parental physical activity and children’s physical activity levels, as well as children’s psychosocial development. Specifically, parental support, beliefs, and behaviors have been identified as important determinants of children’s engagement in physical activity [17,20,21]. From a theoretical perspective, parental behaviors may influence children’s development through processes of socialization and observational learning. Social learning theory suggests that children acquire behavioral patterns by observing significant others, particularly parents, and imitating behaviors that are socially reinforced [14]. In this context, parents who regularly engage in physical activity may serve as behavioral models, shaping children’s attitudes and behaviors related to activity participation and social interaction. Family socialization perspectives further suggest that parental behaviors influence children’s development through both modeling and the opportunities parents provide for engagement in activities that support social and emotional development [9,17]. Several mechanisms may explain how parental physical activity could relate to children’s psychosocial functioning. First, parents who engage in physical activity may create shared activity contexts, such as family play or recreational activities, that promote cooperation, turn-taking, and social interaction [17]. Second, parental physical activity may influence psychosocial outcomes indirectly through children’s physical activity participation, as higher levels of physical activity in early childhood have been associated with improved emotional regulation and social functioning [1,2]. These processes may be particularly relevant during the preschool years, when parents remain the primary socialization agents and children’s social competencies are rapidly developing [6]. Thus, parental physical activity may influence children’s psychosocial development both directly through behavioral modeling and indirectly through increased opportunities for children’s physical activity participation.

However, most empirical studies examining parental influence have focused primarily on two areas: (a) the association between the physical activity levels of parents and their children [22], and (b) children’s social behavior within structured sport or athletic environments [9]. Importantly, the majority of this evidence has been derived from studies involving school-aged children (6–11 years) and adolescents (12–19 years) [22]. Consequently, much less is known about how parental physical activity behaviors may influence both physical activity participation and psychosocial functioning during early childhood. Research focusing on preschool-aged children remains limited, even though early childhood is a critical developmental period for establishing physical activity habits and social competencies. Only a small number of studies have examined the relationship between parental behaviors and preschool children’s physical activity and social behavior [24,25,26], and these studies have often focused on either physical activity or psychosocial outcomes separately. As a result, there is insufficient evidence regarding whether parental engagement in physical activity is associated simultaneously with preschool children’s physical activity participation and key aspects of their social functioning. Therefore, a more comprehensive examination is needed to better understand the role of parental physical activity in shaping both the physical activity behaviors and psychosocial development of preschool children [27]. To address this gap, the present study examined the association between parental physical activity and preschool children’s physical activity participation, as well as indicators of social behavior, including prosocial behavior, hyperactivity/inattention, emotional and behavioral difficulties, and peer relationships. In the present study, parental physical activity refers to parents’ engagement in physical activity during their leisure time. Consistent with previous research on parental physical activity influences on children, the construct includes behavioral indicators of parents’ own physical activity participation, such as the frequency and duration of physical activity during weekdays and weekends, as well as parents’ attitudes or beliefs regarding the importance of physical activity. These dimensions reflect the behavioral modeling component (i.e., parents’ own physical activity behavior) that has been identified as a mechanism through which parents may influence children’s physical activity behaviors (e.g., [22,26] Trost & Loprinzi, 2011; Xu et al., 2015). To improve conceptual clarity, it is important to distinguish between different forms of parental engagement in physical activity. Structured exercise refers to planned, organized, and intentional physical activities performed by parents (e.g., gym exercise, running, or organized sport). General physical activity refers to broader bodily movement occurring in daily life, including activities such as walking, household tasks, or active transportation. Leisure-time physical activity refers specifically to physical activities performed during discretionary time, often together with family members or for recreation. Finally, attitudes toward physical activity refer to parents’ beliefs and perceptions regarding the importance and value of physical activity. Although these dimensions represent distinct but related aspects of the family physical activity environment in the present study, the generic term physical activity was used. Based on previous evidence it was hypothesized that engagement of parents in physical activity will be positively associated with the physical activity levels of preschool children and aspects of the child’s social behavior, such as prosocial behavior, emotional and behavioral concerns, hyperactivity and inattention, and peer relationships.

## 2. Materials and Methods

### 2.1. Participants

The study involved data from 151 preschoolers, comprising 70 girls and 81 boys (Mage = 52.51 months, SD = 3.38). The children were enrolled in private and public kindergarten programs located in the prefectures of Thessaloniki in Greece and Nicosia in Cyprus. While a small cohort of children from diverse national backgrounds existed, the preponderance of the juvenile population was Greek and Greek-Cypriots. The unit of recruitment was the kindergarten teachers. Recruitment of teachers was carried out through a variety of methods, such as online advertisements, distribution of flyers, and personal invitations. The recruitment process involved public and private kindergartens located in Thessaloniki, Greece, and Nicosia, Cyprus. Ten kindergarten teachers agreed to take part in the study. Upon their acceptance, they informed parents about the subject and objective of the study and obtained their consent for their participation.

### 2.2. Measures

The following scales were used to measure the variables under study:

Strengths and Difficulties Questionnaire (SDQ-Hel). The evaluation of students’ psychosocial functioning within the educational setting and broader social context was conducted using the Strengths and Difficulties Questionnaire (SDQ-Hel, refs. [28,29]), which was translated into Greek by Bibou-Nakou, Stogiannidou, Kioseoglou, and Papageorgiou [30]. The survey has a total of 25 items, with each item presenting three response options: ‘not true’, ‘somewhat true’, and ‘definitely true’. The questionnaire comprises a composite difficulty score and five subcategories, each containing five items, namely hyperactivity (e.g., ‘The child is restless, he/she cannot stay still for long’), conduct problems (e.g., ‘The child gets very angry and often lose his/her temper’), peer problems (e.g., ‘The child is usually on his/her own. He/she generally plays alone or keep to himself/herself’), emotional symptoms (e.g., ‘The child gets a lot of headaches, stomach-aches or sickness’), and prosocial behavior (e.g., ‘The child tries to be nice to other people. He/she cares about their feelings’). In this study the informant-rated version was used and was provided by the kindergarten teacher. The score for each of the five scales is made by summing the scores for the five items of this scale, generating a score ranging from 0 to 10.

Preschool-aged children physical activity questionnaire PrePAQ (Home version) [31] was utilized to record the viewpoints of parents concerning their children’s and their own physical activity. In the present study, parental physical activity was operationalized using several indicators derived from the PrePAQ questionnaire, including: (a) frequency of parental physical activity during weekdays and weekends, (b) duration of parental physical activity (minutes spent exercising during weekdays and weekends), and (c) parental leisure-time physical activity. More specifically, the questionnaire has items including: (i) the physical activity levels of parents (reported in times during the weekdays and during the weekends, and minutes spent on physical activity during the weekdays and the weekends) and their parenting beliefs; (ii) demographic characteristics of the family; (iii) the environment within the house and community; and (iv) the innate activity tendencies of the kid. The evaluation of the child’s physical activity encompassed an examination of their engagement in several activities often observed among preschool-aged individuals, with data collected for one weekday and two weekend days. Responses are anchored in a ‘Yes’ or ‘No’; if participants respond ‘Yes’, then the time spent in that activity is requested. Parents reported the number of times usually spent partaking in the activity each week and on weekends and the duration (i.e., minutes) spent partaking in the activity. The physical activity levels of children were categorized into five distinct groups, namely sedentary behaviors (levels 1 and 2), low-intensity physical activity (level 3), moderate-intensity physical activity (level 4), and high-intensity physical activity (level 5). The questionnaire has been validated with preschoolers’ parents [31].

### 2.3. Procedure

The study was conducted in accordance with the guidelines for Ethics in Research of the Aristotle University of Thessaloniki and adhered to the principles outlined in the Helsinki Declaration for research involving human subjects. Informed consent was obtained from all participants (teachers and parents) prior to their participation in the study. Teachers who agreed to participate and signed the consent form were responsible for distributing consent forms to the parents of their students and collecting the completed forms before data collection began. The informed consent document elucidated the objectives of the research, the optional character of involvement, the probable hazards and advantages, and the protocols for safeguarding anonymity and confidentiality. The study’s participants were duly notified that they had the option to discontinue their involvement in the research at any point in time without incurring any negative consequences. In order to maintain confidentiality, any personally identifiable information obtained during the course of the study was segregated from the research data. The removal or substitution of personal identifiers, such as names, email addresses, or social media handles, was carried out through the use of unique codes. To safeguard the anonymity of the participants, any indirect identifiers, such as specific locations or detailed descriptions, were altered. The collected data was restricted to authorized members who were directly involved in the study. The participants were provided with a guarantee that their data would be utilized exclusively for research objectives and would not be divulged to any external parties beyond the research team. The questionnaires were administered to kindergarten educators in hard copy format, whereas parents completed the questionnaires online. Kindergarten teachers completed the SDQHel, whereas parents completed the Pre-PAQ (Home version).

### 2.4. Data Analysis

Statistical analyses were conducted using IBM SPSS Statistics (version SPSS 28). First, descriptive statistics (means, standard deviations, frequencies, skewness, and kurtosis) were calculated to summarize the characteristics of the sample and the main study variables. Internal consistency coefficients were estimated for the multi-item scales. Subsequently, Pearson correlation analyses were performed to examine the bivariate linear associations among the study variables. Consistent with recommendations for quantitative analytical designs [32] (Sánchez-Martín et al., 2024), the analyses were conducted sequentially, beginning with descriptive and correlational analyses to explore relationships among variables, followed by regression analyses to evaluate the associations between parental physical activity and the study outcomes. Prior to the main analyses, the data were screened for extreme values and distributional irregularities, and extreme values were Winsorized to reduce the influence of outliers.

To test the study hypotheses, a series of multiple linear regression analyses was conducted. Separate regression models were estimated for each child outcome variable. Specifically, the psychosocial outcomes included prosocial behavior, emotional symptoms, conduct problems, hyperactivity/inattention, and peer relationship problems, whereas the physical activity outcomes included sedentary behavior (levels 1 and 2), low-intensity physical activity (level 3), moderate-intensity physical activity (level 4), and vigorous-intensity physical activity (level 5). The parental physical activity indicators derived from the PrePAQ (frequency during weekdays and weekends, duration during weekdays and weekends, leisure-time physical activity during weekdays and weekends, and parental beliefs about physical activity) were entered as predictors.

Assumptions for linear regression were examined prior to interpretation of the models. Normality was evaluated using skewness and kurtosis values and inspection of the variable distributions. Multicollinearity was assessed using tolerance and variance inflation factor (VIF) values; no serious multicollinearity problems were identified, as tolerance values were above 0.20 and VIF values were below 5. Given the relatively small number of children nested within each preschool/teacher cluster, the analyses were conducted at the individual level. Statistical significance was set at *p* < 0.05. In addition to statistical significance, effect sizes were evaluated using adjusted R^2^ and R^2^ values for overall model fit and standardized beta coefficients (β) for individual predictors, in order to assess the magnitude of the observed associations.

## 3. Results

Means, standard deviations and normality statistics are presented in Table 1. Extreme values were Winsorized to reduce the influence of outliers. The analysis of correlation revealed moderate correlations among the psychosocial factors and moderate correlations among the physical activity factors (Table 2). Multicollinearity diagnostics indicated no concerns, as tolerance values were above 0.20 and VIF values were below 5 (tolerance levels ranged from 0.200 to 0.753; VIF ranged from 1.32 to 5.00).

Linear regression analyses were performed to investigate the association between parental physical activity and children’s psychosocial development. The results of the regression analysis indicated that none of the parental physical activity variables showed a statistically significant association with positive social behavior (Adj R^2^ = 0.039, F = 1.87, *p* = 0.078). Similarly, no significant association was found for emotional disorders (Adj R^2^ = −0.002, F = 0.956, *p* = 0.466). No significant results were also found about the effect of parental physical activity on conduct disorders (Adj R^2^ = −0.017, F = 0.637, *p* = 0.725). The effect of parental physical activity on hyperactivity and inattention was also insignificant (Adj R^2^ = −0.016, F = 0.669, *p* = 0.698). A non-significant association was also found between parental physical activity and peer relationships (Adj R^2^ = 0.018, F = 1.382, *p* = 0.217). Overall, the models for psychosocial outcomes explained negligible to small proportions of variance (adjusted R^2^ values ranging from −0.017 to 0.039), and none reached statistical significance, suggesting that parental physical activity had little direct association with these aspects of children’s social behavior in the present sample.

With respect to the effect of parental physical activity on children’s physical activity, the regression models explained small to moderate proportions of variance. The model predicting sedentary behavior showed the largest effect (R^2^ = 0.327, adjusted R^2^ = 0.294), indicating a moderate amount of explained variance. Free time activities during the weekends showed a relatively strong positive association with children’s sedentary lifestyle (β = 0.536, t = 5.011, *p* < 0.001), whereas parents’ beliefs about physical activity showed a small negative association (β = −0.188, t = −2.375, *p* = 0.019). With respect to the second level of physical activity, including upper body movement and sedentary activities, such as video gaming, a small positive relation was revealed (R^2^ = 0.126, Adj R^2^ = 0.083, F = 2.930, *p* = 0.007). Parental beliefs about physical activity (β = 0.203, t = 2.237, *p* = 0.027) were the only predictor significantly associated with children’s activity level, with a modest effect size. With respect to low levels of physical activity, the regression analysis revealed a small but significant relation to parental physical activity (R^2^ = 0.116. Adj R^2^ = 0.073, F = 2.683, *p* = 0.012). Free time during the weekdays (β = 0.285, t = 2.071, *p* = 0.040) showed a modest association with children’s low intensity physical activity, whereas time of parental physical activity on weekdays (β = 0.405, t = 2.776, *p* = 0.006) showed a moderate association. In regards to the children’s medium-level physical activity, the results of the analysis indicated a small but significant association (R^2^ = 0.177, Adj R^2^ = 0.136, F = 4.378, *p* < 0.000), with time of parental physical activity during weekends being the only variable significantly associated with (β = 0.415, t = 2.442, *p* = 0.016) with a moderate effect size. Finally, a small but significant association was found with children’s vigorous physical activity (R^2^ = 0.188, Adj R^2^ = 0.148, F = 4.733, *p* < 0.000). Times of parental physical activity during weekends (β = 0.513, t = 3.043, *p* = 0.003) showed a strong relation to vigorous physical activity.

## 4. Discussion

The present study examined the effect of parental physical activity on preschool children’s social behavior and on their physical activity levels. The results of the study provided evidence on the positive association of parental physical activity with children’s physical activity behavior, whereas no significant associations were found for their social behavior at school. With respect to the association of parental physical activity with preschool children’s social behavior, the results of the regression analyses indicated that none of the parental physical activity variables were statistically significantly related to positive social behavior, emotional disorders, conduct disorders, or hyperactivity and inattention. Although several associations with children’s physical activity were statistically significant, the magnitude of the effects was generally small to moderate, suggesting that parental physical activity may be more relevant for children’s movement behaviors than for their broader social functioning in the present sample [25].

One plausible explanation for the absence of substantial associations is that other factors, such as parental approach, familial interactions, or genetic predispositions, might exert a more pronounced impact on these specific outcomes [27]. However, this interpretation should be made cautiously, given the cross-sectional design and the variables included in the present study. Previous studies suggest that parental factors extending beyond physical activity, such as parental warmth and involvement, may also be relevant for children’s social and emotional development [33,34]. Hence, it is recommended that future studies should examine the joint effect of parental physical activity and several other parenting aspects, in order to acquire a more holistic comprehension of their collective relation to the psychological development of preschool-aged children.

A significant correlation between parental physical activity and the sedentary behavior of their children emerged. Engagement in leisure activities on weekends was significantly associated with an increased likelihood of sedentary behavior among children. Conversely, parental attitudes towards physical activity were found to have a negative association with children’s sedentary lifestyle. These findings provide evidence that the level of parental involvement in physical activity has the potential to play a role in children’s sedentary behavior through the promotion or discouragement of sedentary activities during their leisure time. Our findings corroborate those of previous studies that have emphasized the role of parental physical activity behavior and attitudes on the sedentary behavior of children [35,36,37]. In order to foster a more physically active lifestyle among preschool-aged children, it is advisable to urge parents to actively participate in physical activities with their children on weekends, while also cultivating good attitudes towards physical activity. Additionally, it is recommended to limit sedentary activities during leisure time [27].

Furthermore, with regard to the motion of the upper body and activities characterized by a lack of physical activity, such as engaging in video gaming, the statistical analysis demonstrated a noteworthy and meaningful result. The sole significant variable related to children’s level of activity in this group was parental beliefs about physical activity. The results of this analysis indicate that parental attitudes towards physical activity may be associated with the activities that children participate in. Parents who place importance on physical activity and actively participate in it themselves may be more inclined to motivate their children to engage in upper-body movement activities rather than sedentary habits. This observation is consistent with prior studies that underscore the significance of parental role modeling and attitudes in influencing the physical activity habits of children [38,39].

Furthermore, with respect to physical activity of low intensity, the regression analysis demonstrated a significant association with parental physical activity. The study found that free time and parental engagement in physical activity over weekdays were identified as important factors that are related to children’s levels of low-intensity physical activity. Recognizing the need to integrate consistent physical activities into their schedules, parents play a crucial role in promoting the adoption of an active lifestyle among their children, encompassing both weekdays and weekends [24,40]. Our study supports this evidence, with an emphasis on weekdays, and suggests that the physical activity behaviors of parents, as well as the timing of their physical activity sessions, may be related to the level of involvement children exhibit in low-intensity physical activities.

With regard to physical activity of moderate intensity, the regression analysis revealed a statistically significant association with parental physical activity on weekends. Parents may opt to participate in various activities, such as engaging in outdoor play, embarking on family walks, or participating in sports, on weekends. Or even facilitate transportation to physical activity [12]. These endeavors can serve as avenues for their children to engage in physical activities of moderate intensity [41,42] and improve their motor skills [36].

Finally, a noteworthy association was observed in relation to the vigorous physical activity of children. The parental physical activity on weekends was found to be an important determinant. This is consistent with other studies that highlight the beneficial association of parental physical activity with levels of intense physical exertion exhibited by children [38,43,44]. Parents who participate in active play, sports, or other dynamic activities with their children may facilitate their high-intensity physical activity.

### Limitations and Practical Applications

One potential limitation of the study is that the data relied on self-reported measures. Parental responses in this context may exhibit bias as a result of their own subjective assessments of their and their children’s physical activity levels. Similarly, teachers’ responses may have been influenced by their personal ideas concerning psychosocial issues and how these aspects are expressed by the children. Future research endeavors would be enhanced by the utilization of objective metrics to assess physical activity levels, as well as the systematic monitoring of children’s behavior. In addition, in the present dataset, the number of children per preschool/teacher was very small, with several clusters contributing only a few participants. For this reason, the analyses were conducted at the individual level. Nevertheless, it is possible that some residual dependence between observations remains, which should be considered when interpreting the results. Future studies with larger samples within each kindergartner or classroom would allow the use of multilevel analytical approaches to more accurately account for clustering effects. Moreover, the study employed a cross-sectional design that does not allow causal inferences. Future studies should employ longitudinal and intervention designs that can provide meaningful information on the causal relationships between parental physical activity and children’s physical and socio-emotional development. Additionally, the use of self-reported measures may have introduced social desirability bias, potentially leading parents to overreport socially favorable behaviors such as physical activity. Furthermore, the absence of relevant control variables (e.g., socioeconomic status or child characteristics) may have influenced the stability and interpretation of the findings. Lastly, because parents reported both their own physical activity and their children’s physical activity, some of the observed associations may partly reflect shared-method variance rather than exclusively substantive relationships. Notwithstanding these limitations, the results of this study indicate that engaging in physical activity by parents has been found to be associated with their children’s physical activity level.

The findings of this study offer preliminary evidence that can be used to inform tentative recommendations for parents, early childhood educators, and health promotion practitioners. Encouraging parents to engage regularly in physical activity, particularly through shared activities with their children during both weekdays and weekends, may effectively promote higher physical activity levels and reduce sedentary behavior in preschool-aged children. Early childhood educators and policymakers can use these insights to design family-centered interventions that emphasize parental role modeling and positive attitudes toward physical activity. Additionally, integrating opportunities for socially interactive physical activities may support the development of peer relationships in early childhood, highlighting the value of coordinated efforts between families and educational settings to foster active and socially enriching environments for young children.

## 5. Conclusions

The results of this study underscore the significance of parental involvement in physical activity and highlight the necessity for parents to cultivate favorable attitudes. This can be achieved by encouraging an active lifestyle and facilitating opportunities for engaging in physical activities. Moreover, the aforementioned findings underscore the necessity of taking into account many elements that contribute to the psychological development of children and highlight the significance of parental involvement that extends beyond physical activity alone. Further research is required to delve into the dynamics between parental actions, such as physical activity, and other contextual and personal aspects. This pursuit aims to acquire a more holistic comprehension of how these elements collectively impact the psychological well-being of children.

## Figures and Tables

**Table 1:** Descriptive statistics of the study variables.

Variables	Mean	SD	Skewness	Kurtosis	Cronbach α/McDonald Omega
1. Positive social behavior	2.50	0.36	−0.79	0.23	0.79/0.78
2. Emotional symptoms	1.29	0.35	1.24	1.44	0.66/0.66
3. Conduct problems	1.45	0.18	0.95	1.34	0.68/0.70
4. Hyperactivity and inattention	1.82	0.32	0.56	0.30	0.81/0.80
5. Peer relationships	1.85	0.19	0.90	1.30	0.67/0.69
6. Level 1 (sedentary)	27.38	25.94	3.11	13.80	-
7. Level 2 (sedentary)	27.91	22.81	1.11	4.21	-
8. Level 3 (low-intensity)	6.91	6.35	2.82	14.98	-
9. Level 4 (moderate-intensity)	6.47	9.09	3.21	13.03	-
10. Level 5 (high-intensity)	1.18	5.12	4.80	22.69	-
11. Times/weekdays	10.47	8.68	1.97	7.14	-
12. Times/weekend	8.49	6.22	0.89	−0.12	-
13. Minutes/weekdays	333.64	208.40	0.11	−1.44	-
14. Minutes/weekend	273.97	143.15	−0.55	−1.22	-
15. Leisure time/weekdays	237.59	107.83	−0.47	−0.76	-
16. Leisure time/weekend	144.28	86.03	1.42	2.78	-
17. Parental beliefs	3.35	0.36	0.65	0.74	-

Note: Level 1–5 = children’s physical activity (1 = sedentary–5 = high-intensity).

**Table 2:** Correlations among the study’s variables.

Variables	1	2	3	4	5	6	7	8	9	10	11	12	13	14	15	16	17
1. PSB																	
2. Emot_ probl	−0.36 **	1															
3. Cond_probl	−0.18 **	0.22 **	1														
4. Hyperactivity	−0.20 **	0.26 **	0.10	1													
5. Peer relations	−0.13 **	0.31 **	0.23 **	0.02	1												
6. Level 1	−0.00	0.01	−0.09	−0.10	−0.11	1											
7. Level 2	0.23 **	−0.10	0.06	−0.05	0.09	0.18 *	1										
8. Level 3	0.11	−0.10	−0.00	0.03	−0.03	−0.00	0.05	1									
9. Level 4	0.03	.01	−0.02	0.10	−0.04	−0.19 *	−0.21 **	0.38 **	1								
10. Level 5	0.04	−0.03	−0.04	0.06	−0.17 *	−0.18 *	−0.26 **	0.36 **	0.58 **	1							
11. T/weekdays	0.07	−0.01	−0.05	0.06	−0.06	−0.10	−0.27 **	0.20 *	0.34 **	0.24 **	1						
12. T/weekend	−0.00	−0.03	0.00	0.07	−0.09	−0.28 **	−0.19 *	0.06	0.38 **	0.36 **	0.65 **	1					
13. M/weekdays	0.09	0.02	0.03	−0.00	−0.06	−0.00	−0.20 *	0.09	0.24 **	0.25 **	0.77 **	0.56 **	1				
14. M/weekend	−0.11	0.03	0.08	0.03	0.01	−0.22 **	−0.05	0.07	0.22 **	0.18 *	0.38 **	0.75 **	0.47 **	1			
15. LT/weekdays	−0.10	0.13	0.04	0.04	0.05	0.32 **	0.18	0.18 *	−0.15	−0.22 **	−0.23 **	−0.38 **	−0.14	−0.06	1		
16. LT/weekend	−0.07	0.12	0.04	0.08	−0.11	0.48 **	0.07	0.15	−0.07	−0.07	0.05	−0.15	0.15	−0.07	0.68 **	1	
17. P_beliefs	0.16 *	−0.15	0.05	0.03	0.01	−0.30 **	0.02	0.04	0.18 *	0.29 **	0.34 **	0.44 **	0.31 **	0.33 **	−0.33 **	−18 *	1

Note: * = *p* < 0.05, ** = *p* < 0.01; PSB = positive social behavior; Emot_ probl = emotional problems; Cond_probl = conduct problems; Hyperactivity = hyperactivity and inattention; T/weekdays = times/weekdays; T/weekend = times/weekend; M/weekdays = minutes/weekdays; M/weekend = minutes/weekend; LT/weekdays = leisure time/weekdays; LT/weekend = leisure time/weekend; P_beliefs = parental beliefs.

## Data Availability

The data presented in this study are available on request from the corresponding author.
